# Lantern Parades in the Development of Arts in Community Health

**DOI:** 10.1007/s10912-014-9323-3

**Published:** 2014-12-09

**Authors:** Mike White, Mary Robson

**Affiliations:** 1 Research Fellow in Arts and Health, Centre for Medical Humanities, Durham University, Durham, DH1 1SZ UK; 2 Associate Artist for Arts in Health and Education, Centre for Medical Humanities, Durham University, Durham, UK

**Keywords:** Arts, Community, Participation, Social capital, Place, Health promotion

## Abstract

This paper describes the development of two annual lantern parades as case examples of arts in community health, which the authors define as a distinct area of activity operating mainly outside of acute healthcare settings, characterised by the use of participatory arts to promote health. The parades took place in Gateshead 1994–2006 and later in Stockton-on-Tees from 2009 to the present, and the paper reflects on the factors that made for the success of the Gateshead parade and also the problems that led to its demise. It then describes and assesses the Stockton parade, and the benefits and challenges of a workshop ethos of ‘positive regard’ with reference to interview data gathered from adult volunteers and school staff. It considers the potential of this annual ‘tradition’ to shape communal memories that identify with place, and it sets out its aspirations for future programme and research.

This paper presents a collaborative reflection between a researcher and an artist who have been deeply embedded within the arts and community work. It offers a subjective, positioned account of lantern parade work with a view to its future. The authors have been involved in the creation and support of lantern parades for over twenty-five years, increasingly in the context of public health and social pedagogy. This embedded positioning has facilitated research and evaluation through a working practice influenced by the understandings of Rogers about ‘positive regard’ (1959) and Oldenburg’s theory of ‘Third Space’(2000). The key characteristics of ‘Third Place’, as delineated by Oldenburg, are that it provides a neutral ground for non-hierarchical communication and playfulness in a congenial and homely atmosphere. Applying these ideas to our approach has provided us with a philosophically informed pragmatism that frames the work in diverse and challenged communities. Our paper aims to give an informed, affective description of significant steps and processes within our community-based arts in health projects.

At the core of our work is assisting schools and communities to develop new traditions that celebrate health awareness and occasions of transition through resonant imagery and the reflective practice that comes from relationship-based working. This was noted in a contemporary report on a lantern procession in Cumbria in 1988 by theatre company Welfare State International which was the instigator of the earliest of these events:

The procession is the event. It has always been about a process that begins when someone sees the poster, hears the word, and comes to a workshop. That process is complex (involving things social and aesthetic as well as practical) and joyous. The product is the procession; carrying a lantern that you have made through your streets, in front of your friends. The product is by, with and for the procession and its participants.

Such observations were at the root of our subsequent interest to adapt lantern events to health promotion contexts. We realised that lanterns permitted a discreet externalisation of personal feelings within a neighbourhood spectacle, building social cohesion and identity and releasing inner tensions. The open conversations that occurred about health issues in the workshops, the inter-generational working, the personal meanings invested in lanterns, and the beneficial activities intrinsic to this kind of event of a brisk walk and homemade refreshments could provide a microcosm of healthy living.

This paper recounts the development of two annual lantern parades as case examples of arts in community health in Gateshead 1994–2006 and in Stockton-on-Tees 2009 to the present. It identifies the factors that made for the success of the Gateshead parade as well as problems that led to its demise. From lessons learned, it then describes the Stockton parade and the benefits and challenges of a workshop ethos of ‘positive regard’ with reference to interview data from adult volunteers and school staff. It considers the potential of this annual ‘tradition’ to shape communal memories that identify with place, and it sets out its aspirations for programming and research of community-based arts in health events.

## The Wrekenton parade

The *Happy Hearts* celebratory lantern parade for the Wrekenton estate in Gateshead became an annual celebration that took place each March from 1994 to 2006. From the outset the event involved hundreds of local children, their families, voluntary agencies, churches and the district health promotion team. Wrekenton is an area that is regarded as a ‘black spot’ in terms of both its health profile and the media’s image of it as a rough place. Over the years the procession became the distinctive event in the local calendar, a metaphorical ‘screening’ and celebration of community health.

During the 1990s we had felt that we were helping to develop in Wrekenton a model example of practice in community-based arts in health. It addressed both emotional health and physical health through a focus on creating participatory arts activities in a community that presented significant challenges for effective health promotion. A public health report at the time, for example, identified Gateshead borough as having the worst morbidity rate in coronary heart disease in England (Gateshead and South Tyneside Health Authority 1992). From 1994, a group of adult volunteers from Wrekenton estate who were mainly parents of children attending the estate’s primary school became involved with us in developing lantern parades on a healthy heart theme year on year. They were interested in creating this annual event not just to provide some seasonal festivity in the social calendar but also because they grasped its potency as a health promotion tool. They were particularly concerned about evidence that Wrekenton had double the national average of smokers at 43 %, and that a third of young women on the estate smoked. Furthermore, in Gateshead the pre-mortality rates from cancer were among the highest in England at twice the national average. As one resident reflected in an interview with the local paper, the *Newcastle Chronicle Extra* for September 15, 2010, “we wanted to do something to bring down these terrible figures in a way that educated but didn’t patronise people and that would bring the community closer together. A lantern parade started in the mid-1990s to raise awareness of health matters such as heart disease, so it seemed like an ideal way for us to address the issue of smoking.”

When the event began, there was a wealth of networking in Wrekenton. Each year, teachers, health professionals and local artists learned how to make lanterns at an open day held for all interested parties. Reciprocally, the project team were invited to attend the regular local meetings where schools, churches, community education, social services, the youth service, libraries, health visitors and the police were all represented. From these meetings came more support for the project – for example, a second primary school became involved and a teenage mothers group signed up to make images for the procession. A local computer group would produce the poster and the community police officer would organise the route and join us on the parade.

In the project’s last few years, however, the picture became quite different. After 12 years of running a highly successful and influential schools and community project channelling health promotion around the annual sculptural lanterns parade, we had to resign ourselves, for pragmatic and complex reasons, to a reluctant decision that it could not continue. Funding the event had proved more and more difficult, but the biggest concern had been the falling-off of the partner agencies that provided year-round community contact and helped us facilitate a meaningful development of the project as it generated its own narrative from the participants’ perceptions of it. It had become a battle to get the event to happen at all, for lots of reasons, not simply lack of funding. Some of the reasons were that broad community network meetings no longer happened, that changes in schools and agencies meant less of an emphasis on the emotional and social development aspects of the project, that the police started to charge a considerable fee to come on the parade, and that, whilst people from other communities were coming to observe and be involved in the project, it was evident that fewer new local people were signing up to its potential. The shutting down of *Happy Hearts* was felt as a personal disappointment to us as we had embedded our belief in the potency of arts in community health within the spirit of this event.

The Wrekenton parade’s long duration had nevertheless assisted the evolution of our understanding of arts in community health as a distinct area of activity operating mainly outside of acute healthcare settings, characterised by the use of participatory arts to promote health, as described in White’s book on practice and research in this field (2009). The big challenge for arts in community health, it seems to us, has been to sustain projects for long enough to understand and consolidate the practice and to undertake longitudinal research that can utilise and analyse participants’ testimony in a more rigorous ethnographic framework. Inevitably it proved difficult for a parlous community arts project such as the Wrekenton parade, reliant on successive one-off project funds, to maintain the on-the-ground partnerships, attract strategic support and remain vital and engaging within a generational timeframe. For the sustainability of this kind of event, the communal will to be “all in it together” within a climate that generates social capital is crucial.

In our experience of long-term local arts development, lantern processions can provide occasions to view a community in another light, and they can become part of wider programmes of work that connect arts, health education and community development. They can also suggest small-scale but significant practical instances of how social capital is produced and built upon and offer both a tangible image and narrative of how that ‘capital’ is in circulation in the community (White 2009; White and Robson 2011). Some key flaws have been pointed out in social capital theory, however, since the term was first coined by Coleman (1988) McQueen-Thomson and Ziguras (2002) note the term ‘social capital’ has been used almost synonymously with ‘community’ and ‘social networks’ in social health projects literature. It has become a catch-all phrase, loosely defined without recognition that it is a very contested term in sociology and is sometimes used to express the summative outcome of different indicators of benefit. There is also a conundrum in social capital theory as to whether it has demonstrable externalities beyond the value to the individual, making it a communal attribute.

For the community arts sector to address these complexities, it needs to figure more largely in social capital research. Both Marmot (2004) and Wilkinson (2005) have produced persuasive research into the connections between health, status and income, but they do not adequately explain the structure and processes of social support that can mitigate the effects of health inequalities, perhaps because looking at the cultural aspects of this is not in their domain. Likewise Putnam in *Bowling Alone* (2000) focuses more on the level and degree of participation in civil society rather than how and why participatory activities are developed. In a later book, Putnam and Feldstein (2003) recognise, however, the need for a more nuanced approach to the building of social capital, drawing distinctions between ‘bonding social capital’ (ties between people who already have much in common) and ‘bridging social capital’ (ties between people ‘across a greater social distance’). This distinction enlightened our understanding of what happened at Wrekenton and guided us in the formation of the Tilery project, outlined later.

Another, better understanding of the social psychology that goes into building trust and reciprocity in communities is expressed in a book that pre-dates social capital theory, Lewis Hyde’s seminal work on arts and the gift economy, *The Gift* (1979*)*. Hyde contrasts the sterile exchanges of commodity culture with the ability of an artwork or totem to bind a community through an evolving tradition of reciprocal generosity. Making artwork as a social gift is at the heart of thinking and practice in community arts. A gift is not a commodity at all in the sense that its value is perceived wholly in the transmission rather than the accumulation of a good. What matters is the sentiment and ceremony of the process. As Hyde describes it: ‘When a gift passes, it becomes the binder of many wills. What gathers in it is not only the sentiment of generosity but the affirmation of individual goodwill, making of those separate parts a *spiritus mundi*, a unanimous heart, a band whose wills are focused through the lens of the gift. Thus the gift becomes an agent of social cohesion, and this again leads to the feeling that its passage increases its worth, for in social life at least, the whole is greater than the sum of its parts’ (36). This observation has much in common with Richard Sennet’s delineation of the criteria for social inclusion as mutual exchange, ritual and witness to others (1999, 25–27). Hyde emphasises the importance of a process of emotional transaction through creative participation that makes for genuine empowerment rather than a balance sheet deduction of how much social or cultural capital a community may possess. Since the demise of the Wrekenton parade, this has informed our thinking on subsequent lantern events, particularly in respect of the meanings and memories ascribed to them by participants.

The ups and downs of our Wrekenton experience helped us see that there are other overlooked factors that need to be taken into account in the research and evaluation of arts in community health in respect of engagement and sustainability: namely, the resonance within the experience of making artwork, the aesthetic agency of participatory arts, and what we ascribe to be ‘the communal will’ for the continuation of the activity in a ‘traditional’ seasonal pattern. Our logistical problems in sustaining the annual parades in Wrekenton had led us perhaps to undervalue these effects, focusing instead on inputs and outcomes of social capital with a diminishing faith that the community wanted this event because the professional ‘gatekeepers’ had become so uncommitted. These insights have influenced our approach in a more recent programme of work which we shall now describe.

## The Tilery parade

Tilery Primary in Stockton-on-Tees is the nearest school to Durham University’s Queen’s Campus where the School of Health and Medicine is located and is the latest addition to a cluster of schools-based arts in heath programmes in areas of social disadvantage in Northern England that the authors have assisted and/or investigated. Deprivation in Stockton-on-Tees (pop. 83,000) is higher than average and around 9,000 children there are classed as ‘living in poverty’. Life expectancy in the most deprived areas, such as those served by Tilery Primary, is 14.8 years lower for men and 10.4 lower for women than in the least deprived areas of the town. Twenty percent of Year 6 children are obese, and GCSE attainment is lower than the national average (http://www.healthprofiles.info.html).

Tilery Primary draws its school roll from the St. Ann’s and Portrack estates which have seen a significant increase in the last decade of refugees and asylum seekers. The school’s head teacher, John Repton, estimates there are now over twenty languages spoken on the estates, and the growing ethnic diversity is both reflected and addressed in the school. Historically, there has been some hostility between the two estates, and those tensions have been further raised by the influx of ‘newcomers’ perceived as having arrived from afar. A Tilery lantern parade was conceived as one way to bring the community together, to articulate differences and promote tolerance in the changing demographics. The common ground between the communities on the estates is literally a footpath across a strip of common land that connects St. Ann’s to Portrack and over which the parade passes in both directions each year, giving the participants the opportunity to view the full span of the procession before it weaves through each estate. From the comments of participants, this seems to provide a potent image of a joined together community that resonates long after the event.

The Tilery project began in October 2008 when a group of school staff and parents attended Sunderland’s annual ‘Catch the Light’ lanterns event at Southwick school, which the authors have also assisted since 2002. A group of Southwick parents and staff made a gift of a special star-shaped lantern to lead the first Tilery procession in the hope it would provide a tangible and powerfully symbolic message of passing on community knowledge. The first Tilery Lanterns event took place in March 2009, preceded by two weeks of lantern workshops and other health-themed activities in the school. The children’s parents and siblings as well as local voluntary organisations were involved in preparations for the event. Over 90 lanterns were made and around 1,000 people turned out to view the procession, and the enthusiasm of the community for supporting it as an annual event became evident. The second lantern procession in 2010 drew even greater support and Year 6 pupils won a £1,000 prize from the local radio station for providing the most imaginative promotion of its breakfast show – they decorated a large lantern with paper-cut musical notes and a portrait of the programme’s host done by a local tattooist. The 2011 and 2012 parades also saw an exponential growth in participants and spectators and firmly established the event as an annual ‘tradition’. Medical students at Queen’s Campus also started attending the school on a regular basis to engage with the social and emotional learning curriculum as part of their community placements. Furthermore, the nearby Infinity footbridge over the Tees links the Queen’s Campus and the local community, and the whole of Tilery school were invited to cross the bridge soon after it opened in 2009 to meet staff and students and explain the significance of their lanterns event.

Durham’s Centre for Medical Humanities (CMH), where the authors are based, has been able to support the event in its early years through grant awards from the university’s Community Venture Fund and Beacon funds for community engagement in research. A key aim of the partnership has been to establish a community arts and health initiative for the promotion of emotional literacy and emotional health amongst children and the local community of the St. Ann’s and Portrack estates with an accompanying educational aim within Tilery to support children’s aspirations early in life for higher education. This is in keeping with the WHO Global School Health Initiative which states that “the advantages of a positive school environment can be greater well-being and happiness, an improved sense of belonging and better quality of life for those engaged with the organisation” (World Health Organisation 2014).

Crucial to the success of the event each year is the atmosphere generated in the lanterns workshop space, which the authors see as being related closely to the characteristics of ‘Third Place’ community spaces (as distinct from home or work space) defined by Oldenburg (2000). There is an ongoing connection with a current Australian research study into ‘Third Place’ as a conceptual and imaginative environment where learning and creativity combine in community settings. This is a multi-site study of the characteristics of community-based arts in health and is led by Murdoch University with Big hArt (sic) theatre company in Australia, funded by the Australia Research Council. The Tilery Lanterns project has been invited to provide an international comparator for the study. To assess the impacts which accrue and the effective processes used, the study’s four key research questions addressed to participant, community, organisation and funder are:

- What is the range of understandings and expectations in relation to impact?
- What are the diverse forms of evidence required to reveal impact in the field?
- What are the informing principles and practices across the work?
- What benefits and disbenefits accrue from these creative practices?

These research questions have so far not been generally applied across our lantern work but are specific to the context of the Australian research study for which Tilery provides an international comparator. At the 2012 Tilery lantern workshops, some semi-structured interviews, prompted by these questions and closely based on the methodologies used for interviewing in the Big hArt study, were conducted with a small group of parent volunteers and staff. These interviews with participants affirmed the characteristics of ‘Third Place’ that exist in the Tilery workshops. Data gleaned from interviews, project notebooks and albums, digital documentation made by the school and focus-group conversations show that the lantern parades also appear to bring an added depth to the characteristics of ‘Third Place’ with regard to how they assist remembrances, empathic insight, capacity building, and improvements in mental and emotional health.

On the subject of remembrances, and specifically the large star lantern which has been transformed into an armature on which to record lost or absent relatives, one volunteer said, “I liked the Remembrance Star we made this year for who’s here and who’s not. The idea came naturally from us,” and another reported, “I took our lanterns to the cemetery for a memory thing for the kids. It’s important and that’s why we’ve got more remembrances this year. It’s not just sticks and paper, there’s deep meanings.” A focus group that was convened to review the 2012 procession also commented that “The Star of Remembrance worked well. The commemoration made it all the more challenging to do it right. We were surprised by how many remembrances there were. We want to continue this. The community reflects on what has happened and children respond well to it. People were putting poems and their own writing on the stars. Kids connect the light with the remembrance, as though it were enabling the dead person to ‘see’ the event. There aren’t usually opportunities in community and cultural life to do this.”

As regards empathic insight developed through the event, one volunteer said, “It’s about bringing the community together and seeing it. I feel safer because I know people. There was a feeling of strangeness about being with other races at first, but now I’m fascinated by other cultures and the opportunity to be involved through the school. Lanterns show people as they really are and you get an insight into their lives.” Another added, “It’s good to meet all these new people too and have more conversation at the school gates. You get different impressions of people from a lantern workshop and you change your opinion of them.”

Arising from the growing perception of a connected community created through the event, there is a keenness to share the experience with others, establish a network hub for community development and build capacity. As one volunteer noted, “The event has a special meaning for this community and it’s a good opportunity for the university to have an influence on children here,” while another commented, “We need to keep addressing multi-cultural issues and get more of other cultures into the workshops. We should go to other communities and the campus and involve other schools and make it bigger as a town event.” The school’s community liaison worker said, “I want to see us pass this tradition to somewhere else. We shouldn’t keep it to ourselves. Lantern ‘twinning’ would be great. It would be fabulous to link with a community in another part of the world, for example.”

The impact on mental and emotional health was expressed by one volunteer as follows, “When you get your child with you making something, you have a different kind of conversation and make a bond. There’s no stress and it’s good for your health. You can spend time with your child and not worry, you’re both engaged and it eases the burden on yourself. All in all you’re in a better mood. It helps you to understand and deal with your feelings. It gives you time to reflect and get your worries out and not take them home. I can be easier on myself.”

Head teacher John Repton provides the following reflections related to the above quotes:

I wanted our school to make a difference in our community and improve well-being in an area with a poor health profile. I always felt intervention would need to be long-term, aiming to reach the ‘softer’ bits that connect to the kids’ feelings. The arts channel emotional responses to situations [in the community]. They are a vehicle to promote equality, whatever race or background. There’s something special about this event for the community. It has rejuvenated it and it’s welcomed by different generations. Many children say “this is the best day of my life.” We need to keep extending the community focus. The university could help us to become more reflective. We need a higher level of children’s engagement and to develop an interest by the community in local history. We need strong models of good living to keep them on the straight and narrow. We want to transform the school for the development of learning by all the family. There’s a safe community space created through lanterns. Where else can you go for that communal feeling?

There are indications in these comments that staff and parent volunteers at Tilery have come to see the event as providing a space for transformation with a guiding ethos that is mostly clearly articulated in the lantern workshops. The workshops take place over the 2 weeks leading up to the event, the result of planning that has been going on in school since the start of the academic year. School staff are involved at every stage of the project, doing the jobs that need to be done – for example sourcing and buying materials – and also having conversations with children and parents that lead to significant developments such as the large star lantern that has become the focus for community remembrances.

Author Mary Robson and fellow artist Gilly Rogers are the workshop facilitators, working alongside a core group of children’s relatives – largely parents and grandparents - who have become very involved with the project. Workshops happen in school time with the children and then after school and over the weekends when families can attend. School staff, university students on community placements and visitors all get involved in the workshops to varying degrees.

The school has two halls and gives the smaller one over to become the home of the lanterns project for its duration. It is light, with windows down one wall and has the convenience of its own entrance, a nearby kitchen and toilets. It becomes a self-contained hub of activity. At the heart of the workshops is the making of the candle-lit lanterns, from willow sticks, masking tape, tissue paper and glue. Most lanterns are small and house-shaped to represent home and community. Makers include a star shape somewhere in their lantern as a common connecting image. Staff and volunteer teams from local organisations and Year 6 pupils make large lanterns. The processional lanterns have included representations of, for example, a children’s bus from Sure Start, a local church and the Infinity Bridge made by Durham university students.

There is now significant expertise in lantern making in the community. Some Tilery residents have become proficient makers and construct complex sculptural lanterns that demand a lot of commitment and a whole range of decisions in their making. One family made an eight-foot long carp with an elegantly flipped tail. A group made up of two friends and their children surprised themselves by making a stegosaurus the size of a trestle-table. These and other lanterns punctuate the parade with wit, carried by their makers with a real sense of pride, achievement and progress.

The psychologist Rogers (1959) coined the term *unconditional positive regard* to describe what he believed was an essential component of the counsellor/client relationship. The Tilery facilitators place *unconditional positive regard* at the heart of relationship-making during the workshops. They attempt to show complete support and acceptance of a person no matter what that person says or does. On the whole, this helps the workshops be positive experiences. Importantly, it also helps to articulate difficult scenarios: for example, when one refugee family made a mosque lantern, a racist comment was posted on a social networking site by another participant and was brought to the attention of school staff and the facilitators. The team took the opportunity to have a conversation with the core group of adult volunteers who were due to take on the running of a Saturday workshop. Without naming names, there was an in-depth discussion about the incident and what it meant for the event. People could safely voice their opinions and feelings and meaningfully contribute to the communally-held beliefs about the event. The feelings of resentment and anger that were bubbling under surfaced. One result was that a relative of the perpetrator took to Facebook in defence of the communal nature of the parade, and all offensive remarks were taken down from the site. The perpetrator has since re-joined the event, and the problem appeared to have been solved through honest reflection, without having to point a judgemental finger.

So the workshop space becomes one where anything can be talked about and where meaning is made. Putnam and Feldstein (2003) quote the words of a Chicago mayor, Harold Washington, that are inscribed in the floor of the public library: “All have come together to mix and contend, to argue and to reason, to confront our problems and not to contain them.”

It is easy for these lantern events to be viewed as relentlessly positive, but the path to the parade is strewn with problems that have to be confronted and not merely contained. Here are some examples:

- Participation can be limited by circumstance--for example, the child who is desperate to attend but who doesn’t because his carers haven’t participated and so do not see any value in attending.
- As people are more encouraged to make their own individual lanterns, there is a need to maintain a communal sense of what is appropriate – for example, the large Union Jack lantern made in good faith but that could have sent out the wrong message to community members.
- In a space that is bridging great social distance, respectful lines have to drawn– for example, comments made about lifestyle choices of participants.
- As happened at Wrekenton, local organisations are suffering cut-backs and don’t prioritise the parade. For example, Sure Start staff no longer build a lantern.

The culture of the Tilery parade is that facilitators, staff and participants variously work together to solve the problems and make the tricky decisions. So when one local organisation is unable to attend, another takes its place. Developments such as eating lunch together on the weekend sessions, home-cooked by the head, school staff and children add to the cohesiveness of the project and encourage further communication between all participants.

Participants work together to consciously create a shared history. Project artists, staff, children and participants document daily events. Here are extracts from posts co-written by Mary Robson and participants from the blog of the final day of the 2012 event (www.dur.ac.uk/cmh):

The Tilery Team is a big one – artists, school staff, pupils and their families – and we all roll along together, getting things done. Today’s tasks included Years 5 and 6 covering their large lanterns and helping younger children get theirs finished; a host of parents and families finishing their creations; covering the Star of Remembrance and affixing the messages that have been collected.

Appropriately enough, Mrs. Robinson, a Yr 6 girl and I worked on the latter at the end of the day, when the workshop was quieter than it had been all day. We gently glued the messages on, one by one, carefully and respectfully reading them as we went. Some anonymous, some generic, some poignantly specific. This lantern will be carefully sited in the yard – it won’t be taken on the parade but will stand sentinel until we return.

…at the workshop, Gilly and the ladies were soldiering on, finishing and covering. We had a mountain of work in front of us, but everything stopped for tea and for lunch. Extra help arrived as the day went on – researchers from the Centre for Medical Humanities, three medical students and two artists – and all were folded in to the mix. The atmosphere was calm and purposeful and deeply enjoyable; we knew we would get there and were confident enough to leave for fish and chips in the staff room at 4.20 pm.

There is a purposeful, collaborative atmosphere that builds in the last couple of days. Everyone who arrives can find a place, be ‘folded in’ to the experience. Here seems to be instinctive team work.

As the parade returns to the school, all the lanterns are collected together. They form a tableau with all the small lantern houses in a semi-circle around which everyone gathers once they have their well-earned hot chocolate. Within the semi-circle, the larger lanterns are arranged so that all can be seen. In 2012, this tableau became more significant. The Star of Remembrance was central – it was there waiting for the parade to find it. A four-year-old pupil who had died after being struck by a car on the estate was commemorated by a crown shaped lantern, made by his family for their ‘little prince’, carefully positioned in the middle of the space. The crowd hushed as three young cousins of the boy stood behind the crown and sang a song in his memory and as a comfort to his family. Then the choir led us all in what has become the usual rousing rendition of “This Little Light of Mine, I’m going to let it shine.” Here, there is an atmosphere of togetherness and a job well done. Also, there is the feeling that the image of the lanterns all together has to be absorbed, enough to last for another year, until the next time.

The shaping of a communal memory of the lanterns event is crucial to the solidarity that sustains the repetition of the event in the social calendar. Francois Matarasso’s case study account of amateur participatory arts in *Where We Dream* (2012) notes, “Shared memories are a cornerstone of community. They are shorthand through which people reconnect with – and renew – past feelings. They bind us together” (27).

## Next steps

Tilery primary and the community it serves are fully committed to the continuation of the annual lanterns workshops as a means of improving community well-being and integration, and the authors would now like to assist them through a next phase of development titled *The Tilery Chronicles*, which will build the momentum of current activities and introduce new ones. We hope to develop our understanding of social capital beyond ‘networks of trust and reciprocity’ as defined by Coleman (1988) and Putnam (2000) and look at how nurtured conversation around these activities may generate communal memories and shared meanings, interpreted across the diversity of the community.

*The Tilery Chronicles* will test a hypothesis that exploring the local history of the neighbourhood and its social and economic relations outwards can build temporal, or vertical, community connectedness. This will complement how through the annual lantern parade the building of a local cultural tradition can formulate through narratives a spatial, or horizontal, community connectedness. The next phase of research will draw on a range of art forms and interventions to connect the history of the area, of families and of newcomers. It will support the intergenerational collection of personal stories and communal journals, create ‘suitcase stories’ for children in transition from primary to secondary school, support family events in the school and build historical aspects into the community’s awareness of its changing identity.

The inspiration and content of *The Tilery Chronicles* is to be drawn from the local history of the area and from the heritage of local people and their widely differing cultural backgrounds. The project also aims to establish community enterprise through the school that expresses its ethos of inclusion – e.g. developing specialty bread-baking for events in a seasonal calendar. The Centre for Medical Humanities at Durham wants to introduce reflective practice in the school and community to inform the research, and this has already commenced through participatory documentation of the annual lanterns procession presented in blog entries on ‘Tilery’ (http://www.dur.ac.uk/cmh.html).

On the issue of sustainability, although individual arts in community health projects may come to an end, there is rarely a final outcome from this relationship-based way of working because it is always ongoing. This is why the narrative of a project becomes so important because, as the ‘new public health‘pioneer Michael Wilson asserted, health requires “the languages of story, myth and poetry [to] also disclose its truth” (1975, 60–61). Much of what is described earlier in this paper in the case examples is informed by the narratives of the participants and their perception of producing new traditions for their community through the event. This suggests to us that ethnographic accounts informed by medical humanities might be the most effective and sympathetic means of understanding the practice of arts in community health and assessing its benefits. It would permit researchers to immerse in the processes of how culture and well-being inter-relate.
